# Alectinib and lorlatinib function by modulating EMT-related proteins and MMPs in NSCLC metastasis

**DOI:** 10.17305/bjbms.2020.5066

**Published:** 2021-06

**Authors:** Xu Feng, En-Shi Xu

**Affiliations:** 1 Department of Neurosurgery, The First Affiliated Hospital of Jinzhou Medical University, Jinzhou, Liaoning 121002, China; 2 Department of General Surgery, The First Affiliated Hospital of Jinzhou Medical University, Jinzhou, Liaoning 121002, China

**Keywords:** ALK, alectinib, lorlatinib, VIM, FN1, MMPs

## Abstract

Most advanced non-small cell lung cancer (NSCLC) patients are accompanied by brain metastasis which is the major cause of increased mortality. The fusion rearrangement of anaplastic lymphoma kinase (ALK) gene is an important feature of brain metastasis in lung cancer. The novel ALK inhibitors alectinib and lorlatinib are shown to be effective against NSCLC brain metastasis, while their underlying mechanism of action is unclear. Epithelial–mesenchymal transition (EMT) proteins and matrix metalloproteinases (MMPs) play important roles in brain metastasis by regulating the blood-brain barrier (BBB). To reveal the molecular function of alectinib and lorlatinib, we explored their effects on the cellular levels of EMT markers: VIM and FN1 and the matrix metalloproteinases MMP-9 and MMP-7. The mRNA and protein levels of VIM, FN1, MMP-9, and MMP-7 were elevated in H3122 cells. However, upon alectinib and lorlatinib treatment, the levels were significantly reduced. Similar results were obtained when these experiments were performed either in a dose-dependent or time-dependent manner. Furthermore, alectinib and lorlatinib also inhibited the cell viability and migration of H3122 cells. Interestingly, in comparison to individual drugs, the combination of alectinib and lorlatinib was found to be substantially more effective. Overall, these results suggest that alectinib and lorlatinib possibly function through the downregulation of MMPs and EMT in NSCLC metastasis.

## INTRODUCTION

Lung cancer is a grave public health concern worldwide [1]. Among all the cancers, it is ranked first in men and among the top three in women based on high incidence and mortality rate. The rates are even further rising [2]. The majority of cases (about 85%) of lung cancer is non-small cell lung cancer (NSCLC) [3]. Of these, 3-7% of NSCLC is due to the rearrangement of the carcinogenic anaplastic lymphoma kinase (ALK) gene. Unfortunately, most of these patients later submit to brain metastasis which is the leading cause for higher mortality [4]. The prognosis of brain metastasis is poor and the median survival time of untreated brain metastases may be only 7 months, even though the median survival time of untreated brain metastases is generally <3 years [5].

The key event of brain metastasis is the passage of lung cancer cells through the blood-brain barrier (BBB) [6,7], epithelial-mesenchymal transition (EMT) related proteins [7], cell adhesion molecules [6], and matrix metalloproteinases (MMPs) play important roles [8]. During the EMT, epithelial cells attain the mesenchymal phenotype. This leads to the disruption of tight intercellular junctions that facilitate cell migration. The upregulation of *fibronectin* 1 (FN1) and *vimentin* (VIM) genes is considered as the molecular marker for mesenchymal transition. Interestingly, FN1 and VIM are also known to play regulatory roles in NSCLC brain metastasis [9]. Similarly, MMPs are the enzymes that function by degrading the extracellular matrix proteins to expose the sites for cell invasion. MMPs such as MMP-7 and MMP-9 have been found to promote tumor invasion in NSCLC metastasis [10].

At present, the novel ALK inhibitors alectinib and lorlatinib are shown to be effective against NSCLC brain metastasis [11-13]. However, the underlying molecular mechanism of their action is still unclear. It would be interesting to know if alectinib and lorlatinib inhibit NSCLC metastasis by regulating MMPs and/or EMT events. Understanding such a regulation would expand our knowledge of alectinib and lorlatinib treatment in NSCLC patients.

In this study, using quantitative real-time polymerase chain reaction (qRT-PCR) and western blot analysis, we first analyzed the effect of alectinib and lorlatinib on the expression and protein levels of FN1, VIM, MMP-7, and MMP-9. Furthermore, we carried out the CCK-8 assay and scratch experiments to determine their effects on cell viability and mobility, respectively. We suggest that ALK inhibitors alectinib and lorlatinib function by downregulating the MMPs and EMT genes in NSCLC metastasis.

## MATERIALS AND METHODS

### Materials

Alectinib and lorlatinib were purchased from Selleck (Shanghai, China). CCK-8, RPMI-1640 mediums, Dulbecco’s Modified Eagle’s medium (DMEM)-H mediums, and PBS were purchased from Thermo Fisher Scientific Inc. (Shanghai, China). RIPA lysis buffer and apoptosis kit were obtained from Nanjing Jiancheng Bioengineering Institute (Nanjing, China). NSCLC cell line H3122 and human bronchial epithelioid cell line 16HBE were acquired from Nanjing Cell Bank of the Chinese Academy of Sciences (Nanjing, China). The antibodies were obtained from Abcam Inc. (USA).

### Cell culture

H3122 cells were cultured in RPMI-1640 medium containing 10% fetal bovine serum (FBS) and 1% penicillin-streptomycin at 37°C and 5% CO_2_. Similarly, the 16HBEcells were cultured in DMEM-H medium supplemented with 10% FBS and 1% penicillin-streptomycin at 37°C and 5% CO_2_.

### qRT-PCR

Specifically, the total RNA from the sample was extracted and transcribed into the corresponding cDNA using a reverse transcription kit. The qPCR reaction was conducted using SYBR qPCR Detection Kit. The qPCR program began with the initial 3 minutes of denaturation step at 95°C to stimulate the hot-start i TaqTM DNA polymerase followed by 45 cycles of denaturation at 95°C for 10 seconds. Annealing and extension were at 60°C for 45 seconds. The *β-actin* gene was used as an internal reference.

### Western blot analysis

The total protein samples were isolated, and the concentration measurements were carried out. The extracted protein samples were then separated on SDS-PAGE gel and later were transferred to the nitrocellulose membrane. The membrane was activated using methanol. About 5% of skim milk in TBST was used to block the nonspecific interactions. The blocked membranes were later incubated overnight with corresponding primary antibodies. After the washing steps, incubation with secondary antibodies horseradish peroxidase-bound immunoglobulin G was carried out. The signal was measured by the detection of enhanced chemiluminescence. The β-actin was used as a loading control.

### CCK-8 assay (cell viability assay)

Cells were inoculated into 96-well plates (5 × 10^3^ cells/well) and cultured for 24 hours, 48 hours, 72 hours, and 96 hours, respectively. At indicated time points, cells were further incubated with 10 μl of CCK-8 reagent for another 2 hours at 37 °C. The absorbance was detected using a microplate reader at 450 nm.

### Scratch experiment (cell migration assay)

1 × 10^7^ cells were inoculated into a 6-well plate and cultured under normal conditions. When cells were in the logarithmic growth phase, a 100 μl gun head was used to scribe lines in the well plate, and the pictures were recorded under the microscope. Subsequently, the normal culture medium was replaced with the culture medium containing alectinib and lorlatinib alone or in combination, and the growth was further allowed for 24 hours. This was again photographed under a microscope for analysis.

### Statistical analysis

SPSS 19.0 statistical software (Chicago, IL) was used for statistical analysis. Results are expressed as mean values ± standard deviation. Analysis of variance was performed to compare the data among the groups. All the experiments were repeated 3 times. *p* < 0.05 was considered to indicate a statistically significant difference.

## RESULTS

### Alectinib and lorlatinib reduce the expression of EMT-related proteins and MMPs

**FIGURE 1:**
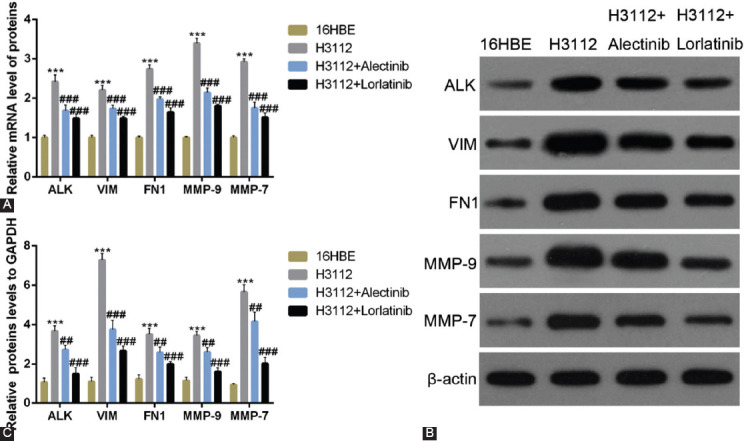
Alectinib and lorlatinib reduce the expression of cell adhesion molecules and matrix metalloproteinases. (A) The mRNA expression of anaplastic lymphoma kinase (ALK), vimentin (VIM), fibronectin 1 (FN1), MMP-9, MMP-7 in H312 cells treated with 0.1 μM alectinib or 0.1 μM lorlatinib. (B and C) The protein expression of ALK, VIM, FN1, MMP-9, and MMP-7 in H312 cells treated with 0.1 μM alectinib or 0.1 μM lorlatinib.

To assess the effect of alectinib and lorlatinib on the expression of EMT marker proteins and MMPs, qRT-PCR experiments were carried out. The results showed that the mRNA expressions of VIM, FN1, MMP-9, and MMP-7 were increased in H3112 cells. However, upon 0.1 μM alectinib and 0.1 μM lorlatinib treatment the mRNA expression levels of these got significantly reduced (Figure 1A; ***p < 0.05, ###*p* < 0.01, vs. 16HBE). When western blot analysis was carried out to measure the protein levels, results showed that the protein levels of VIM, FN1, MMP-9, and MMP-7 were also reduced by 0.1 μM alectinib and 0.1 μM lorlatinib in H3112 cells (Figure 1B and C; ***p < 0.05, ###*p* < 0.01, vs. 16HBE). Notably, in both the experiments, we found that compared to alectinib, the effect of lorlatinib was slightly higher. The β-actin was used as the internal loading control for western blot analysis. The protein and mRNA levels in 16HBE cells were uses as a baseline.

### Alectinib and lorlatinib reduce the expression of EMT-related proteins and MMPs in a dose-dependent manner

**FIGURE 2:**
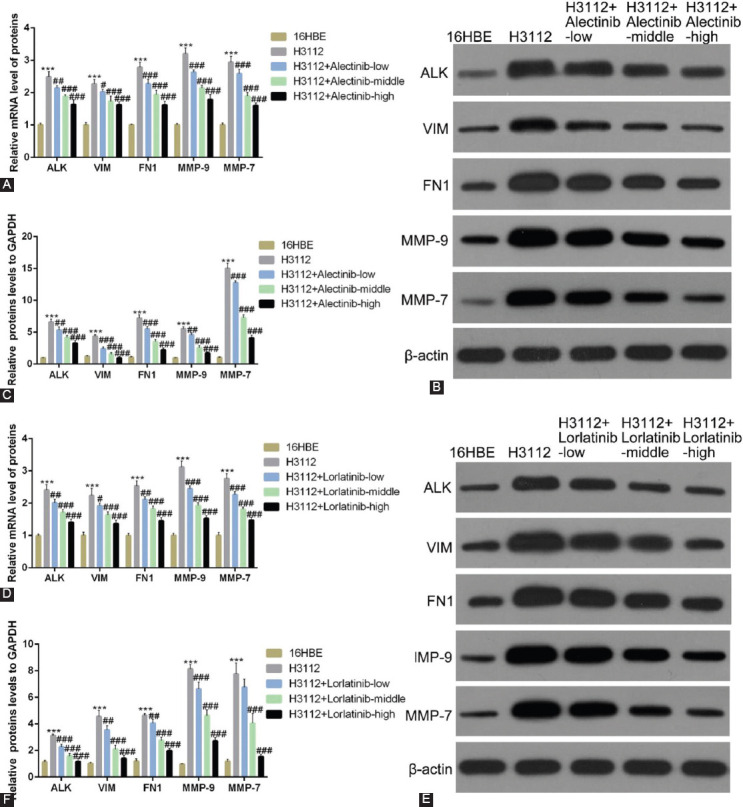
Alectinib and lorlatinib reduce the expression of cell adhesion molecules and matrix metalloproteinases in a dose-dependent manner. (A) The mRNA expression of anaplastic lymphoma kinase (ALK), vimentin (VIM), fibronectin 1 (FN1), MMP-9, MMP-7 in H312 cells treated with alectinib in different doses (0.01 μM, 0.1 μM, and 1 μM). (B and C) The protein expression of ALK, VIM, FN1, MMP-9, and MMP-7 in H312 cells treated with alectinib in different doses (0.01 μM, 0.1 μM, and 1 μM). (D) The mRNA expression of ALK, VIM, FN1, MMP-9, and MMP-7 in H312 cells treated with lorlatinib in different doses (0.01 μM, 0.1 μM, and 1 μM). (E and F) The protein expression of ALK, VIM, fibronectin 1, MMP-9, and MMP-7 in H312 cells treated with lorlatinib in different doses (0.01 μM, 0.1 μM, and 1 μM).

Similar to the experiments performed in Figure 1, dose-dependent experiments were carried out. Here also, qRT-PCR showed that alectinib dose-dependently decreased the mRNA expressions of VIM, FN1, MMP-9, and MMP-7 in H3112 cells (Figure 2A; ***p < 0.05, ###*p* < 0.01, vs. 16HBE). Similar results were obtained when western blot analysis was performed. The protein levels of VIM, FN1, MMP-9, and MMP-7 were also suppressed by alectinib in a dose-dependent manner (Figure 2B and C; ***p < 0.05, ###*p* < 0.01, vs. 16HBE). Interestingly, Figure 2D, qRT-PCR showed that lorlatinib also decreased the mRNA expressions and protein levels of VIM, FN1, MMP-9, and MMP-7 in a dose-dependent manner in H3112 cells (Figure 2E–F; ***p < 0.05, ###*p* < 0.01, vs. 16HBE).

### Alectinib and lorlatinib reduce the expression level of EMT-related proteins and MMPs in a time-dependent manner

**FIGURE 3:**
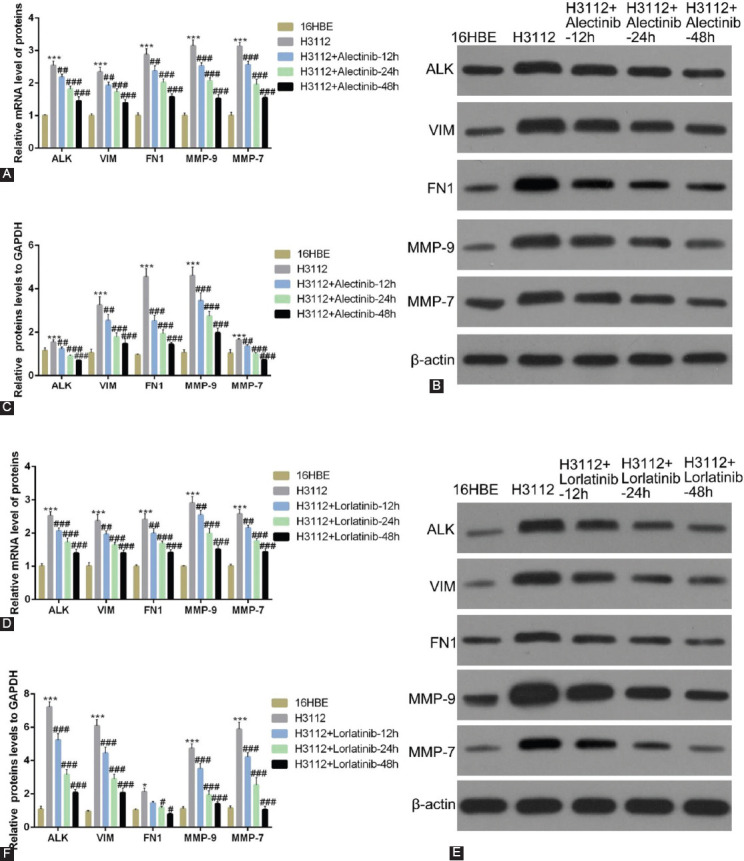
Alectinib and lorlatinib reduce the expression level of cell adhesion molecules and matrix metalloproteinases in a time-dependent manner. (A) The mRNA expression of anaplastic lymphoma kinase (ALK), vimentin (VIM), fibronectin 1 (FN1), MMP-9, and MMP-7 in H312 cells treated with 0.1 μM alectinib at different time points. (B and C) The protein expression of ALK, VIM, FN1, MMP-9, and MMP-7 in H312 cells treated with 0.1 μM alectinib at different time points. (D) The mRNA expression of ALK, VIM, FN1, MMP-9, and MMP-7 in H312 cells treated with 0.1 μM lorlatinib at different times. (E and F) The protein expression of ALK, VIM, FN1, MMP-9, and MMP-7 in H312 cells treated with0.1 μM lorlatinib at different time points.

To further strengthen the findings, we next carried out time-dependent experiments. The results from qRT-PCR revealed that the mRNA expressions of VIM, FN1, MMP-9, and MMP-7 were significantly reduced by 0.1 μM alectinib in a time-dependent manner in H3112 cells (Figure 3A; ***p < 0.05, ###*p* < 0.01, vs. 16HBE). Similarly, in western blot analysis too, 0.1 μM alectinib decreased the protein levels of VIM, FN1, MMP-9, and MMP-7 in a time-dependent manner (Figure 3B and C; ***p < 0.05, ###*p* < 0.01, vs. 16HBE). When comparable experiments were carried out using lorlatinib, qRT-PCR data show that 0.1 μM lorlatinib too decreased the mRNA expressions of VIM, FN1, MMP-9, and MMP-7 in a time-dependent manner in H3122 cells (Figure 3D; ***p < 0.05, ###*p* < 0.01, vs. 16HBE). Furthermore, western blot results too showed that protein levels of VIM, FN1, MMP9, and MMP7 in H3112 cells were decreased by 0.1 μM lorlatinib time-dependently (Figure 3E and F; ***p < 0.05, ###*p* < 0.01, vs. 16HBE, vs. 16HBE).

### A combination of alectinib and lorlatinib is significantly more effective than a single drug treatment

**FIGURE 4:**
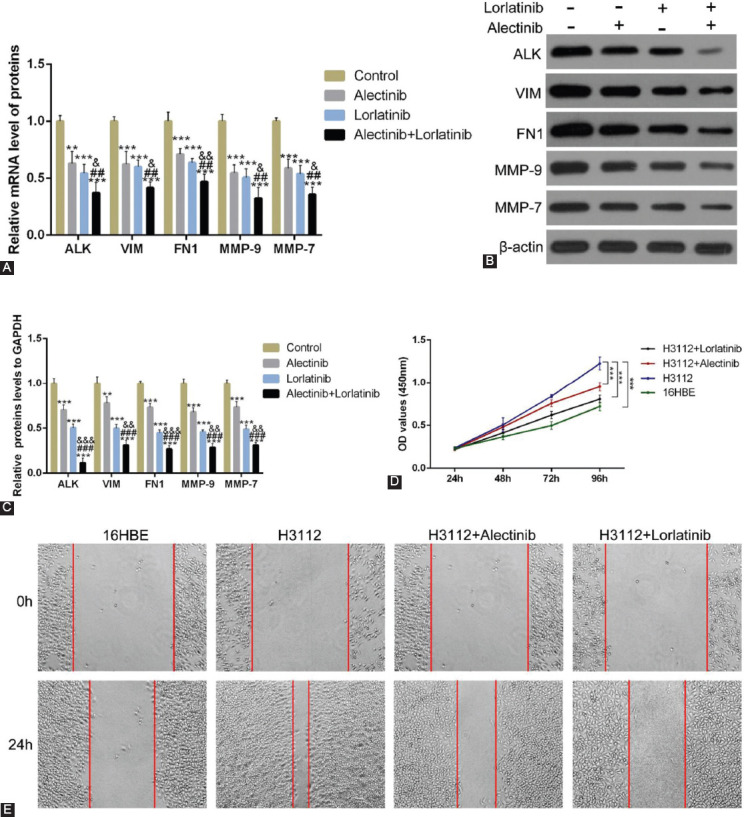
Alectinib combined with lorlatinib significantly reduces the expression of cell adhesion molecules and matrix metalloproteinases. (A) The mRNA expression of anaplastic lymphoma kinase (ALK), vimentin (VIM), fibronectin 1 (FN1), MMP-9, and MMP-7 in H312 cells treated with 0.1 μM alectinib and 0.1 μM lorlatinib. (B and C) The protein expression of ALK, VIM, FN1, MMP-9, and MMP-7 in H312 cell treated with 0.1 μM alectinib and 0.1 μM lorlatinib. (D) Cell viability of H312 treated with 0.1 μM alectinib or 0.1 μM lorlatinib. (E) Scratch experiments of H312 treated with 0.1 μM alectinib or 0.1 μM lorlatinib.

After establishing the individual effect of alectinib and lorlatinib, we further tested if a combination of both drugs would have a synergistic effect. For this, we carried out the experiments using drugs in isolation and combination. Interestingly, the results from qRT-PCR showed that the mRNA expressions of VIM, FN1, MMP-9, and MMP-7 decreased significantly after a combination treatment of 0.1 μM alectinib and 0.1 μM lorlatinib (Figure 4A; ***p < 0.05, ###*p* < 0.01, and *p* < 0.001, vs. control) Western blot analysis too showed similar effect and the combination treatment significantly decreased the proteins levels of VIM, FN1, MMP-9, and MMP-7 (Figure 4B and C; ***p < 0.05, ###*p* < 0.01, *p* < 0.001, vs. control).

Next, we carried out the CCK-8 cell viability assay of H3112 cells. In this assay, too, we found that though the individual drugs also reduced the cell variability, the combination of two drugs was significantly more effective (Figure 4D; ***p < 0.05, vs. 16HBE). When we carried the scratch experiment to test the cell migration of H3112 cells, here also compared to control samples though the individual drugs were effective, the co-treatment of alectinib and lorlatinib was more effective (Figure 4E). Interestingly, similar to the results seen in Figure 1, we noticed that in comparison to alectinib, the effects of lorlatinib were slightly better.

## DISCUSSION

Lung cancer is the most common cancer due to poor prognosis and high incidence rate [14]. The majority of lung cancers are diagnosed at an advanced stage with a higher rate of brain metastasis [15]. At present, the clinical therapy for lung cancer is limited and the curative effects are not satisfactory [16,17]. The majority of lung cancer belongs to NSCLC, where a rearrangement in the *ALK* gene plays a positive role in 3%-13% of cases [18,19]. Therefore, clinicians are now paying substantive attention to the *ALK* gene for diagnosis and treatment of NSCLC. The treatment method largely focuses on the use of drugs alectinib [20]. However, within a year of treatment, drug relapse is seen due to a variety of resistance in most patients. Furthermore, it shows poor penetration to the central nervous system and therefore fails to target brain metastasis in NSCLC. Recently, alternative drugs, the novel ALK inhibitors alectinib and lorlatinib have been found promising for the treatment of brain metastasis in NSCLC [11,12]. However, their molecular mechanism is not clearly understood.

The ability of the drug to cross the BBB and then to remain in the brain tissue is highly dependent on its physicochemical parameters such as size, liposolubility, interactions with plasma proteins, and other similar factors [15]. In the case of NSCLC brain metastasis, ideally, the anti-tumor drugs need to pass the BBB, even before the blood supply system of lung cancer metastasis has been established. It is almost impossible to prevent the metastasis spread once its blood supply system is established and the BBB gets completely compromised [4]. Interestingly, EMT-related proteins and MMPs have been shown to play important roles in the invasion and brain metastasis [7].

EMT is an important step in cancer metastasis that leads to increased permeability of cancer cells [21]. E-cadherin, N-cadherin, VIM, twist1, snail, and FN1 are the molecular biomarkers of EMT [22]. The *VIM* gene encodes for the member of intermediate filament family protein mostly found in the mesenchymal cells. This protein helps in maintaining cell shape, cytoplasm integrity, and cytoskeletal interactions. It, along with microtubules and actin microfilaments, creates the cytoskeleton and also found to be involved in the immune response. Interestingly, it also regulates the transport of low-density lipoprotein cholesterol for esterification. It organizes several other critical proteins that are involved in attachment, migration, and cell signaling. The *FN1* gene encodes for FN protein, a glycoprotein that is present as the soluble dimeric form in plasma and as a dimeric or multimeric form at the cell surface and in the extracellular matrix (ECM). FN functions in cell adhesion and migration, metastasis, and other cellular processes such as embryogenesis, wound healing, and blood coagulation. We found that the protein level and mRNA level of these molecules decreased significantly after the addition of alectinib and lorlatinib (*p* < 0.05). Therefore, the combination of alectinib and lorlatinib can significantly reduce the metastasis of lung cancer cells.

In the normal physiological processes, such as embryonic development, reproduction, and tissue remodeling, the MMP family of proteins performs the breakdown of ECM. Interestingly, during metastasis, these get upregulated to perform a similar role. Most MMPs are secreted as inactive proproteins and get activated upon cleavage by extracellular proteinases. The enzyme MMP-9 degrades type IV and V collagens. It is also suggested to involve in IL-8-induced mobilization of bone marrow hematopoietic progenitor cells in the rhesus monkey. In murine, MMP-9 has been shown to play a role in tumor-associated tissue remodeling. The other MMP, MMP-7 degrades FN, elastin proteoglycans, and casein. Peculiarly, it differs from most other MMP family of proteins due to lack of a conserved C-terminal domain. MMP-7 is involved in wound healing and suggested to regulate defensins proteins in the intestinal mucosa.

## CONCLUSIONS

The role of EMT biomarkers FN1 and VIM and the matric metalloproteinases, MMP-9 and MMP-7, in lung cancer in the background of ALK mutation is under study. However, it was important to investigate if the action of alectinib and lorlatinib on NSCLC metastasis was mediated by EMT-related proteins and MMPs. To test that, we evaluated the levels of EMT-related proteins and MMPs in alectinib and lorlatinib treated NSCLC cells. In this study, we found that alectinib and lorlatinib significantly reduced the expression of VIM, FN1, MMP-9, and MMP-7 and that finally inhibited the metastasis of lung cancer cells. Furthermore, we found that in our study though lorlatinib was slightly better than alectinib, the combination of the two was even more effective. These results provide new insight into the mechanism of how the drugs work. However, the mechanism of alectinib and lorlatinib on other biomarkers and important regulators of NSCLC brain metastasis would demand further study.

Since this study is conducted on cell lines, clinical trials are needed to introduce this treatment into the standard. Therefore, we will increase patient samples for clinical experimental research in the future.
